# Building research capacity in child welfare in Canada

**DOI:** 10.1186/s13034-016-0103-x

**Published:** 2016-06-14

**Authors:** Nico Trocmé, Catherine Roy, Tonino Esposito

**Affiliations:** School of Social Work, Philip Fisher Chair in Social Work, McGill University, 3506 University Street, Room 303, Montreal, QC H3A 2A7 Canada; Centre for Research on Children and Families, McGill University, 3506 University Street, Room 106, Montreal, QC H3A 2A7 Canada; Canadian Research Chair in Social Services for Vulnerable Children, School of Social Work, Université de Montréal, 3150 Jean-brillant, Montreal, QC H3T 1N8 Canada

**Keywords:** Evidence-based practice, Partnership, Research-capacity building, Research utilization, Youth protection

## Abstract

There is a surprising dearth of information about the services provided to the children and families being reported to Canadian child welfare authorities, little research on the efficacy of child welfare services in Canada, and limited evidence of new policies and programs designed to address these changes. This paper reports on a research capacity building initiative designed to address some of these issues. By fostering mutual co-operation and sharing of intellectual leadership, the *Building Research Capacity* initiative allows partners to innovate, build institutional capacity and mobilize research knowledge in accessible ways. The model rests on the assumption that by placing the university’s research infrastructure at the service of community agencies, robust research partnerships are developed, access to agency-based research is significantly enhanced and community agencies make better use of research findings which all equate in greater research utilization and research capacity building.

## Background

### Trends in rate of maltreatment and out-of-home care

The primary source of information on child welfare services in Canada is the Canadian Incidence Study of Reported Child Abuse and neglect, a cyclical survey that has been conducted every 5 years at the provincial or national levels since 1993 [1, 2]. At the national level, the study has documented a dramatic increase in child maltreatment investigations, expanding rapidly from a rate of 21.47 investigations per 1000 children in 1998 to 39.16 in 2008 [3]. A second source of information are provincial annual reports on the number of children placed in out-of-home care: combing these reports over the past 20 years, Jones and colleagues [4] reports that the rate of children in care has increased steadily from 5.7 children per 1000 in 1992 to 8.5 per 1000 in 2013. While international comparisons must be made with caution, all indications are that Canada has one of the highest rates of out of home placement amongst countries with fully developed national child protection systems [5].

Analysis of the CIS reports shows that the increase can be attributed primarily to increased reports from professionals about cases of risk of maltreatment and of children exposed to intimate partner violence, but that there has been no change in the number of seriously injured children [6]. Furthermore, a decreasing portion of these investigations actually lead to youth protection services, dropping from 35 % of investigations leading to service in 1998 to 27 % in 2008 [3]. Despite the scope of these changes, there is a surprising dearth of information about the services provided to the children and families being reported to Canadian child welfare authorities, little research on the efficacy of child welfare services in Canada, and limited evidence of new policies and programs designed to address these changes. This paper reports on a research capacity building initiative designed to address some of these issues.

### Child welfare data

In Canada, data on service provision, service trajectories and service outcomes are currently limited. Provincial and federal child welfare information systems have not been used to generate the kind of data required to understand service trajectories, to identify outcomes or to evaluate the impacts of community risk level factors. In fact, most jurisdictions are unable to report on basic information such as the stability and duration of out-of-home placements, academic achievement, rates of recurrence or recidivism or even the extent to which families have access to parenting, substance abuse or mental health services. The CIS is actually the only national source of data on child welfare services. Though informative on many aspects, data from the CIS is cross-sectional and is only designed to produce national estimates.

Even when longitudinal data are available, it is not always easy for agencies to use it. Youth protection organizations in Quebec for example have access to the best developed client and service information system in the country yet its use is limited mainly because of agencies’ under-developed research capacity. Consequently, youth protection agencies in Quebec currently do not have the capacity to analyze their own service statistics beyond providing standard reports based on month-end or year-end activity counts.

Numerous factors explain why child welfare agencies generally have limited research capacity. For one, unlike health sector disciplines, social services do not have a strong research culture. Access to academic journals is very limited for most youth protection agencies making it very challenging to use recent research results to inform clinical practice or program design. Second, infrastructure of most agencies is not suited to promote a strong research culture. Most child welfare agencies do not have researchers or statisticians on staff and very few have standard procedures to review proposals from external researchers. Third and finally, the specificities of the child welfare context (crisis ridden families, ethical issues with children and urgency of protection taking precedence) dissuades many researchers from engaging in research with agencies.

### Child welfare research

In the mid 1970s, Kammerman and Kahn [7] conducted a review of the state of knowledge with respect to the effectiveness of residential and foster care programs and they concluded that not only was there a lack of systematic data, but that practice decisions were essentially based on value judgments and assumptions. More than 25 years later, services to children and families too often continue to be provided irrespective of evidence of service effectiveness. Every year across Canada, over 200,000 children and youth, a rate of 39 per 1000, come into contact with child welfare services authorities [3] and on any 1 day of the year, over 65,000 children and youth are living in out of home care [4]. Despite these large numbers, we know surprisingly little about the children, youth and families involved in these services, and even less about the efficacy of child welfare services. In a report on early childhood development, the Royal Society of Canada claims that “despite consistent evidence of the severe and long-lasting effects of child maltreatment, research on how to best intervene to prevent maltreatment and its recurrence is surprisingly limited” [8]. Even with a growing expectation that the administration of social services should be outcome driven and evidence-based rather than solely needs based [9–12], the field’s evidence, particularly regarding effective practice techniques in core child welfare services remains at an early stage [13]. Compared with other disciplines like pediatric health, education or criminology, child welfare and youth protection research is lagging behind in terms of evidence-based practice [14]. As such, frontline workers continue to struggle to assist children and families, either because too little evidence is available on how to help them best cope with the complex realities they live in or because the evidence-based knowledge that exists is not suited to those realities [15].

In the health and mental health sectors, evidence-based practice was initiated by a knowledge mobilization movement led by Drs. Guyatt and Sackett from the evidence-based medicine working group [16]. First seen as incongruent with the practice of “good” medicine, costly and time-consuming, there is now reliable evidence of improved care. The basic principle of evidence-based medicine, that we should treat where there is evidence of benefit and not treat where there is evidence of no benefit (or harm), is also of relevance for the field of child welfare and youth protection, but only recently has it been given considerable attention. A well-fitted illustration of this is the recent research on foster care which shows that removal of children from their homes, once thought as a protective measure, actually does not ensure that the children will eventually do better than those who remain at home [17]. That is not to say that foster care places children at additional risk of doing poorly but rather, it has not yet proven to improve children’s lives [17].

Because the safety and well-being of children they serve can depend on it, child welfare agencies are, more than ever, being held accountable for the achievement of positive outcomes [13, 18, 19]. As mentioned by Berliner and colleagues [20], when services are mandated rather than sought voluntarily, as is the case in Canadian child welfare, as well as in other child-protection oriented countries, there is an increased ethical duty to ensure that the mandated services have the promise of delivering the intended benefit and ideally, can do so efficiently and without unnecessary burden. There is thus a growing consensus within the field that one of the critical need for youth protection agencies and workers is to ensure evidence is used effectively [18]. But the road to evidence-based practice within high-stake fields such as child welfare is not without obstacles. Evidence-based practice is a multistep process that involves changes both at the individual and organizational levels, which are dependent on thoughtful planning, agency cohesion and workers’ engagement.

### Promoting evidence-based practice

Evidence-based practice, or evidence-informed practice as some authors prefer to refer to, has been put forward as a way of promoting future research initiatives, improving links to policy and creating an organizational learning culture that supports critical thinking and practice and that is firmly rooted in evidence but nonetheless grounded in the realities of practice [21]. At the core of evidence-based practices are the notions of critical thinking, reflective practice and research-based decision-making [22, 23]. On a more concrete note, evidence-based practice involves, but is not limited to, conducting sound assessments, identifying specific measurable intervention goals, monitoring progress toward the attainment of these goals, using critical thinking to select the most effective intervention, assessing whether or not positive outcomes have been achieved and finally, training service providers so that they have the skills and knowledge to meet all of these goals [24].

Needless to say that the challenge for child welfare agencies is substantial given the many resources that are needed to support the activities likely to promote evidence-based practice. One of the priority condition highlighted in the literature is the ability of staff to use and analyze data that is available [25–27]. As highlighted by Aarons and Palinkas [28], if workers are able to utilize research and data efficiently, it makes them informed research customers who are better able to critically appraise research outcomes as well as better skilled at identifying actions needed to promote better outcomes. In Canada, we cannot assume that most front line workers know how to use, make sense of and critique data. Despite efforts to revamp the social work curriculum, most Canadian social workers have a low level of training in research or quantitative analysis. As such, agencies need to be willing to put supports in place that teach workers how to access data, use it, analyze it and modify practices accordingly.

Currently, there is a tendency for provincial governments to impose performance-management agendas that meet their accountability needs without necessarily capturing the outcomes that are viewed as most relevant by agency managers and workers [29, 30]. Authors argue that this “adoption” style of applying empirical research findings in the field in which outcomes are compliantly reported solely to fulfill preset goals, has to yield place to a more “developmental” style of evidence-based practice, which lay its foundations into the practitioner’s desire to facilitate beneficial outcomes for the client [26]. This echoes Hall’s conclusions which critique traditional approaches that exclude practitioner knowledge and client perspectives and minimize the contextualization of research evidence [31]. In fact, because of the many challenges youth protection faces in using evidence-based research and translating it into evidence-based practice, many authors have concluded that evidence-based practice in its purest form, focusing primarily on adopting the findings of empirical research, is not adapted to the reality of child welfare [32, 33]. Rather, they argue that a broader array of variables need to be considered within evidence-based practice, including but not limited to findings from empirical research [31]. That is, knowledge needs to be built from both a “research-based” practice and a “practice-based” research, recognizing that both empirical and reflective evidence can contribute to better outcomes for the children and families child welfare services work with. To take on Fielding and colleagues [33] words, practitioners must come to envision themselves not only as knowledge-takers but also as knowledge makers.

### Bridging research and practice

In an earlier paper, Trocmé [17] has argued that the development of an evidence-based approach within child welfare services is complicated by the fact that there is little consensus, and many contradictions, about the objectives of child welfare services. Family preservation of child protection? Child well-being or child safety? Child protection or family and community support? There is no simple nor unique way of addressing these conflicting objectives. Child welfare is complex domain and finding the most appropriate framework to address it is not an easy task. Traditionally, practitioners, administrators and researchers have worked in silos, turning to outcome measurements for different purposes. Much data has been generated on prevalence but little on actual outcomes. Tools and programs have been conceptualized and implemented but rarely evaluated. In the haste of developing much needed data, measures and interventions, key actors in the field of child welfare may have undermined the development of valid and innovative ways of conducting research. Successful implementation of novel approaches in child welfare research is conditional to overcoming two main challenges namely, the participation of agency staff and managers to research activities and the ability of these same people to conduct data analysis using the administrative data they have access to.

### Participatory data analysis

Participatory research is designed to break down the researcher-subject hierarchy by including communities where the research is taking place as equal partners in the research process [34]. Borda Fals [35], a leading researcher in the field of participatory action research sums up well the guiding principles of conducting participatory research when he says: “*Do not monopolize your knowledge nor impose arrogantly your techniques, but respect and combine your skills with the knowledge of the researched or grassroots communities, taking them as full partners and co*-*researchers (…) Do not depend solely on your culture to interpret facts, but recover local values, traits, beliefs, and arts for action by and with the research organizations. Do not impose your own ponderous scientific style for communicating results, but diffuse and share what you have learned together with the people, in a manner that is wholly understandable and even literary and pleasant, for science should not be necessarily a mystery nor a monopoly of experts and intellectuals*.”

Despite its growing popularity in child welfare, traditional participatory research does not integrate quantitative data analysis methods. Yet, to achieve a level of evidence-based practice that rests on the ability to monitor progress and outcomes using agency data requires skills that current child welfare workers do not have [24]. Indeed many of the analytic methods required to understand service trajectories require a degree of training that could pose a barrier to the inclusion of non-specialists at the analytic and interpretation stages. For example, child welfare managers and policy analysts rely primarily on month-end and year-end cross-sectional service statistics to describe children in out-of-home care. While such counts provide an accurate measure of the number of placements used on any 1 day, they fail to distinguish between short-term and long-term placements. Compared to a longitudinal analysis, such cross-sectional counts tend to significantly over-estimate long-term placements; in a typical child welfare agency about half of all placements on any 1 day are long-term, yet only 10–15 % of all children placed in out of home care end up in a long-term placement. Cross-sectional statistics are also unable to track key information such as movement in care, placement and duration of placements.

Using a participatory data analysis approach which combines the general principles of participatory research with the use of statistical methods typically used in traditional investigator driven research [36, 37], it is possible to train managers to understand and even use a range of longitudinal and multivariate analytical methods. Earlier experiences have demonstrated that by doing so, it is possible to provide a much richer picture of service trajectories and outcomes, including length and stability of placements, patterns of recidivism and involvement in the child welfare system [38]. From the onset, the process of jointly interpreting data allows stakeholders to provide context, insight and recommendations throughout so the research team understands what is most meaningful to them. Within a management context, client and service delivery data can be more efficiently compiled and analysed than is possible through formal researcher driven projects. This in turn increases the likelihood that the project outcomes will be applied in practice [37, 39]. Eventually, the approach is also beneficial to a capacity building process where stakeholders gain the analytical skills they need to set their own research priorities for long-term sustainability [39].

The shift toward the use of research evidence in practice marks an important turning point in a field in which practitioners have traditionally been at best, little engaged in and at worst, separated from academic research. The *Building Research Capacity initiative* (BRC) described below goes one step further by deploying researchers to support agency based analysis team so as to create a model of participatory research that is rooted both in evidence-based practice and quantitative analysis.

### The building research capacity initiative

BRC is a Social Sciences and Humanities Research Council (SSHRC) funded knowledge mobilization and capacity development partnership between academic researchers affiliated with McGill’s Centre for Research on Children and Families (CRCF) and community partners. These include four mainstream youth protection (YP) agencies, a First Nations (FN) youth protection agency and two province-wide service associations representing mainstream and FN service providers. BRC has been developed to support youth protection organizations’ capacity to use clinical, administrative and population statistics to understand better the service trajectories and outcomes for the children, youth and families they serve. The initiative supports formal partnerships between academic researchers, businesses and other partners that advances knowledge and understanding on critical issues of intellectual, social, economic and cultural significance. By fostering mutual co-operation and sharing of intellectual leadership, the initiative allows partners to innovate, build institutional capacity and mobilize research knowledge in accessible ways. The BRC model rests on the assumption that by placing the university’s research infrastructure at the service of community agencies, robust research partnerships are developed, access to agency-based research is significantly enhanced and community agencies make better use of research findings which all equate in greater research utilization and research capacity building. Graduate student (BRC trainees), supported by a team of university researchers, work as knowledge brokers with community agencies by providing a range of support services, from accessing and summarizing studies from academic journals, to designing questionnaires for internal client or staff surveys, to developing data capture tools, to analyzing data, to writing proposals and reports. In most instances these information gathering, analyzing and synthesizing projects remain the property of the community agencies and are used for their administrative purposes.

### BRC objectives

Research capacity building is the foundational objective on which the BRC initiative rests. Operationally, it is translated through two core components namely the use of administrative data and the training of students and agency staff.

### Use of administrative data

One of the focus of the initiative is to support the use of service statistics as a program planning and management tools. To do so, the BRC initiative capitalizes on the untapped potential of administrative datasets in Quebec which contain detailed service information on over 500,000 children who have been involved with youth protection agencies since the mid 1980s. Profiles of children and families, including forms and severity of maltreatment, family structure, selected child and parent characteristics, services provided, types and duration of placements, court involvement as well as census track level community characteristics are available providing a unique opportunity to go beyond the usual descriptive, cross-sectional analyses.

The Service Statistics Information Groups (SSIG) are responsible for directing all analyses, from the selection of questions, to developing operational definitions, to interpreting findings and reporting results within their organizations. SSIGs are agency-specific in that they focus on priorities set by each agency. SSIGs’ members are fully engaged in the analytic process, attending meetings approximately every 6 weeks to define questions, develop operational definitions, interpret and contextualize results and identify additional avenues for analysis. Between meetings, the research team and the agency’s IT specialist develop definitional and analytic options that are then brought back to the SSIG for discussion. The SSIGs also direct activities related to reporting and disseminating the results of the most pertinent analyses.

### Use of research

Another focus of the BRC initiative is to promote the development of a stronger research utilization culture. Moving beyond the focused management driven questions that drive the SSIGs, the initiative also offers participating organizations support from Clinical Integration Groups (CIGs) designed to help clinical staff and managers “keep up with the literature”. CIGs are a combination of a journal club and a clinical expert discussion group. They are organized around a specific area where staff have developed clinical expertise, for example child sexual abuse or intimate partner violence. Their overall purpose is to promote integration of research knowledge and clinical expertise. Sources of knowledge include the literature, research findings, clinical experience as well as administrative data. Members are expected to be self-motivated, clinically driven and interested in furthering their own professional development and connecting knowledge to practice with respect to a particular clinical area. The groups meet approximately every 6 weeks and can include 12–15 members, including managers and clinicians representing various points of service in an agency. Members can also include community experts and stakeholders that would facilitate an information sharing process. The CIGs are led by two agency co-chairs who are responsible for the identification and selection of participants as well as the overall operation of the group. Each CIG is supported by a knowledge brokering team including a university-affiliated researcher who has expertise in the clinical area, as well as a research assistant who provides support for the group’s activities. This team model provides an opportunity for the researcher to engage with clinical experts at the agency, and for the research assistant, a student interested in the area, to support the researcher and the group by conducting literature searches, obtaining articles, and keeping minutes.

### Other training and research-related activities

Over the course of its 6 year duration, the BRC initiative will recruit three cohorts of graduate students, each for 2 years of training. In total, approximately 30 graduate students will be trained over the course of the initiative, added to that number the many researchers and agency workers or managers that will also join the various training components of BRC. The training curriculum integrated to the BRC initiative is indeed extensive and builds on many skills, knowledge and abilities all likely to promote research capacity both at the individual and organizational levels. Table 1 provides a summary of the many activities that have been put in place since the beginning of the initiative with a short description for each.

**Table 1:** Training and research-related activities of the BRC initiative

Activity	Description
Quantitative data analysis	Intensive seminars and semester-long courses focusing on quantitative research methods, from descriptive statistics to multivariate models. Seminars and courses offered up until now have focused on data analysis using SPSS, descriptive and inferential statistics, multilevel models of analysis and statistical analyses using the CIS database
Qualitative data analysis	Seminars and workshops focusing on introductory qualitative research methods including the fundamentals of qualitative interviews, focus groups and data analysis and coding
Child welfare policy group	The group is designed to help students develop and share their expertise on emerging child welfare policy and practice issues across Canada
Journal watch	This group is designed for students and researchers to discuss ongoing child welfare research trends by analyzing and appraising the methodology and substantive content of assigned articles. Articles include ones that stand out because of their methodological strengths or their relevance for policy and practice
Research seminars	Seminars provide an opportunity for students to learn about emerging research in child welfare from other scholars and discuss issues related to research methods or designs, as well as implications for practice and policy
Methods brownbag	A forum to present methodological questions including complex issues of research design, measurement and data analysis
In the know	Newsletter about agency-specific analyses of children’s trajectories

## Conclusion

Despite the promising outlooks of evidence-based practice on outcomes for children and families receiving youth protection services, there is currently little evidence on the extent to which child welfare staff actually engage in it [25]. Even when workers demonstrate a desire to engage in evidence-based practice related activities, few of them are actually able to use the data efficiently, either because the data is not organized in an appropriate manner, not available or because the workers lack the necessary training to use it in a useful manner. In a study conducted on 551 American workers from either public or private child welfare agencies, a quarter of respondents indicated that they rarely came together as a team to discuss or evaluate the effectiveness of their interventions. In the same study, only a third of staff reported they had access to data to help them understand the impact of their work on clients [40]. Based on the knowledge we have of the Canadian context typical of child welfare, it is fair to assume the Quebec and Canadian realities are no different with regards to the use of data to promote evidence-based practice.

There is a growing field of studies that focus on implementation and improvement of methods to promote the systematic uptake of research findings and EBP into community service settings [41]. More and more models highlight the critical role of research-community partnerships to support the relevance and organizational “fit” of interventions to maximize uptake and to build organizational infrastructures to support intervention sustainability [43]. Compared to traditional research-driven models, it is reasonable to believe that these models have “*the potential to improve the utility of interventions, the success and efficiency of uptake, the sustainability of interventions in targeted services and the ultimate effectiveness with clients*” [43] but more research is needed to fully grasp the impact of community-research partnerships on evidence-based practice. Further investigation is needed to identify if practitioners involved in such partnerships do achieve a competent level of evidence utilization and if this improved level of competence increases the likelihood that the desired outcomes for children and families are achieved as well [44].

The BRC initiative has been put in place as a way to promote evidence-based practice by providing the agencies and the individuals the necessary toolbox to interpret, critique, use and conduct evidence-based research. Indeed, beyond its substantive contribution in terms of analyzing service trajectories and outcomes for children involved with youth protection services, the BRC initiative also provides a novel opportunity to test the application of quantitative data analysis methods using a participatory research approach in a youth protection context. Beyond providing training and supporting the development of a stronger evidence based management and practice culture, the initiative will also assist managers in developing or adapting information management tools to allow them to continue to track service trajectories using the data queries developed through the project. It is expected that the shared commitment of resources, shared responsibilities in governance as well as a shared sense of opportunity or benefit provide the foundation for ensuring that this initiative will have a sustainable impact [45]. Through their involvement in the SSIGs, data exchange conferences, the CIGs and data analysis workshops, agency staff will develop the skills to continue to analyze and interpret service statistics in order to better understand and improve the services provided for children, youth and families deserved by youth protection services. The impact of the BRC initiative is being tracked through a process and outcome evaluation. Expected outcomes for this initiative include (1) an overall increase in child welfare productivity as documented by new research projects and citation counts for dissemination outputs, (2) an increased use of research in participating agencies as demonstrated by reference to research in agency documents, expectations that program decisions be evidence-based and a stronger research culture across all hierarchical levels and (3) an increased research capacity as demonstrated by improved research skills for students and staff, and increased agency time allocated to research related activities. In the literature, frequent and sustained interactions such as the ones promoted within the BRC initiative have been found to be important components in building capacity over time [46]. Preliminary evidence does suggest that practitioners who are competent in evidence-based practice have skills for appraising and applying evidence, extensive knowledge of available evidence, use evidence in their practice and possess positive attitudes towards evidence-based approaches [47]. Observations and conclusions following the actualization of the BRC initiative will with no doubt contribute to this field of knowledge and hopefully lead the way to other promising initiatives likely to impact positively on children and families.
